# SunSmart? Skin cancer knowledge and preventive behaviour in a British population representative sample

**DOI:** 10.1093/her/cyh010

**Published:** 2005-01-11

**Authors:** A. Miles, J. Waller, S. Hiom, D. Swanston

**Affiliations:** ^1^Health Behaviour Unit, Cancer Research UK, Department of Epidemiology and Public Health, University College, London WC1E 6BT and ^2^Communications Department, Cancer Research UK, 61 Lincolns Inn Field, London WC2A 3PX, UK

## Abstract

The incidence of skin cancer has risen rapidly in the UK over the last 20 years, prompting public health organizations to try and raise awareness of the dangers of sun exposure and the need to practice sun-safe behaviour. This study aimed to assess baseline levels of sun-safe knowledge and behaviour in a British population-representative sample, prior to the launch of Cancer Research UK's ‘SunSmart’ campaign. A face-to-face survey was conducted through the Office for National Statistics as part of their Omnibus survey. In total, 1848 men and women aged 18 and over were interviewed. Knowledge of what to do to reduce skin cancer risk was modest. Two-thirds mentioned avoiding the sun by seeking shade, 50% mentioned covering up and only 43% said to use high factor sunscreen. Practice of sun-safe behaviours was also poor, with only one-third saying they sought shade, covered up or used high factor sunscreen to protect themselves from the sun. Men and those from lower socioeconomic groups were least informed and least likely to report using sun-protective behaviours. Increases in both knowledge and use of appropriate sun-protective behaviours are needed if skin cancer incidence rates are to decrease.

## Introduction

Skin cancer is currently the most common form of cancer in the UK and rates of the more serious type (malignant melanoma) have doubled in the last 20 years (Quinn *et al.*, 2001). The majority of skin cancers can be prevented by avoiding exposure to UV radiation (International Agency for Research on Cancer, 1992), and numerous countries have launched public awareness campaigns in an effort to reduce skin cancer incidence and mortality rates (Goldsmith *et al.*, 1996; Health Education Authority, Department of Health and British Association of Dermatologists, 1995; Montague *et al.*, 2001).

In Australia, such campaigns have been run for more than 20 years, and have been credited with the high levels of skin cancer awareness and sun-safe behaviour now evident there (Marks, 1999; Montague *et al.*, 2001). Similar efforts within the UK, though, have been more sporadic. The last national campaign began in 1995, but ended in 2000 with the demise of the Health Education Authority. Subsequently, Cancer Research UK was awarded funding by the government to run a nationally coordinated ‘SunSmart’ campaign.

The present study was conducted within the context of the SunSmart campaign to assess baseline levels of sun-safe knowledge and behaviour prior to its launch, and to establish whether the current skin cancer prevention campaign still needed to raise awareness or should focus on changing attitudes and behaviour. Although studies into skin cancer knowledge and sun-safe behaviour have been conducted in the UK (Fleming *et al.*, 1997; Darling and Ibbotson, 2002), few have reported comprehensive data on a general population sample. Whilst a population representative survey was conducted a few years ago (Department of Health, 1998), this report is now out of print and so detailed information on its findings is no longer in the public domain.

## SunSmart campaign

Campaign strategy for SunSmart was agreed by the UV Health Promotion Group, whose members include representatives of the National Radiological Protection Board, British Association of Dermatologists and UK Health Departments. It was launched in March 2003 and is due to run until at least 2007.

Burning and sun exposure during childhood are both significant skin cancer risk factors (Whiteman and Green, 1994; Whiteman *et al.*, 2001), and multiple strategies for avoiding sun exposure are recommended, such as avoiding the sun, covering up and using high factor sunscreens (Vainio *et al.*, 2000; Weinstock, 2001). The main messages of the campaign therefore centre on five key actions people should take to protect against UV damage, presented under the acronym: SMART.

- **S**tay in the shade 11 a.m.–3 p.m.
- **M**ake sure you never burn
- **A**lways cover up with a T-shirt, brimmed hat and wraparound sunglasses
- **R**emember to take extra care with children
- **T**hen use factor 15+ sunscreen

## Methods

Cancer Research UK commissioned a survey through the Office for National Statistics (ONS) as part of their Omnibus survey in February 2003. This baseline research was designed to measure:

- Awareness of sun protection campaigns.
- Knowledge about sun protection behaviours.
- Perceived importance of known skin cancer risk factors.
- Attitudes to tanning and the sun.
- Self-reported practice of sun protective behaviour.

All the questions were developed by the authors to assess the impact of the campaign, and were piloted to check for readability, comprehension, face and content validity. There were limited ways of further validating the items, because financial constraints meant only single-item measures could be used.

Face-to-face interviews were conducted in people's homes by trained interviewers. Background information including age, sex, occupation and education was collected to allow differences between demographic groups to be explored.

### Sample

The sampling frame was the Postcode Address File of those classified as ‘small users’, which includes all private household addresses in England, Scotland and Wales. A sample of 3000 addresses was randomly selected, and stratified for region, the proportion of people renting their home from local authorities and by socioeconomic group (Office for National Statistics, 2003). Of the 3000 addresses selected, 2768 were eligible (i.e. were private households rather than business addresses or new/empty properties). One person was identified from each of the 2768 eligible households. Twenty-five percent of people approached to take part declined and 8% could not be contacted. In total, 1848 of the 2768 people selected to take part were interviewed, giving a response rate of 67%.

Fifty-four percent of the sample were women and the mean age was 49 years. The majority of respondents (56%) were married or living as married and 30% had children living in their house. Approximately one-third had no educational qualifications and 87% owned their own home either jointly or singly. Thirty-one percent had jobs classified as managerial and professional.

### Knowledge

An open-ended question was asked about sun protection ‘What actions should you take to reduce the risk of skin cancer?’. Answers were categorized by the interviewer (i.e. they were not shown to the respondents): only the responses categorized as ‘Stay in the shade/move out of the sun’, ‘Cover up (with long sleeves/trousers, a hat etc)’, ‘Use high factor sunscreen/suntan lotion (factor 15+)’ and ‘Use any sunscreen/suntan lotion’ are reported here.

The perceived importance of known skin cancer risk factors was assessed with the question: ‘How important do you think the following are in increasing the risk of skin cancer?’ and participants were asked to rate each of the following: ‘Sun bathing using suntan lotion’, ‘Sun bathing without using suntan lotion’, ‘Using sunbeds’, ‘Having had sunburnt skin in childhood’, ‘Getting sunburnt as an adult’, ‘Having a large number of moles on your skin’ and ‘Having fair skin with freckles that burns easily’. Response options were ‘Very important’, ‘Quite important’, ‘Not very important’ and ‘Not at all important’.

### Attitudes

Two positive and two negative attitude statements were developed to reflect commonly cited advantages and disadvantages of sun exposure (Arthey and Clarke, 1995). ‘How much do you agree or disagree with the following statements?’: ‘A suntan makes me look more attractive’ ‘A suntan makes me look healthier’, ‘My skin will age more quickly if I spend time in the sun’ and ‘I am concerned that exposure to the sun/UV may give me skin cancer’. Response options were ‘Strongly agree’, ‘Agree’, ‘Neither agree nor disagree’, ‘Disagree’ and ‘Strongly disagree’.

### Behaviour

Participants were asked: ‘Do you personally do anything to protect yourself from the sun and/or skin cancer?’: ‘Yes’, ‘No’ and ‘Don't know/Not sure’.

If participants answered ‘Yes’ they were asked an open-ended question ‘What do you do?’ and again their responses were categorized by the interviewer. Only the responses categorized as ‘Stay in the shade/keep out of the sun’, ‘Cover up (general)’, ‘Wear a hat’, ‘Wear a long sleeved shirt/blouse or long trousers/skirt’, ‘Use a high protection sunscreen/suntan lotion’ and ‘Use sunscreen/suntan lotion (factor unspecified)’ are reported here. Participants were also asked ‘Do you burn easily in the sun?’ and responses were coded: ‘Yes’, ‘No’, ‘Don't know/Not sure’.

### Data analyses

Logistic regression was used to establish the independent predictive effects of demographic variables on knowledge, the perceived importance of skin cancer risk factors, attitudes and behaviour.

## Results

Respondents' knowledge, attitudes and behaviour related to sun exposure, and the sociodemographic predictors of these factors are shown in Table I.

**Table I.** Skin cancer knowledge, attitudes and behaviour among a British population representative sample (frequencies and sociodemographic predictors within logistic regression)

	Percent	*N*	Sociodemographic predictors (Wald statistic)										
			Age	Gender	Children in household	Educational qualifications	Marital status	Burn easily in the sun
Knowledge of what actions to take to reduce the risk of skin cancer (% who know, unprompted)																
high factor sunscreen	42.7	790	50.14^a^^,^^b^	12.51^a^^,^^b^	1.33^a^	19.31^a^^,^^b^	11.72^a^^,^^b^	3.57^a^
stay in shade	64.7	1196	25.41^a^^,^^b^	11.37^a^^,^^b^	0.23	12.85^a^^,^^b^	0.01	0.31
cover up	49.7	919	13.17^a^^,^^b^	0.20	1.27^a^	24.33^a^^,^^b^	0.50^a^	9.76^a^^,^^b^
Skin cancer risk factors endorsed as ‘quite’ or ‘very’ important																
sunbathing without suntan lotion	94.0	1651	2.90	5.38^a^^,^^b^	3.13^a^	5.93^a^^,^^b^	0.03	8.91^a^^,^^b^
sunbathing using suntan lotion	61.5	1034	6.36	1.23	1.11	2.04	1.92^a^	4.31^a^^,^^b^
getting sunburnt as an adult	87.5	1492	2.01	17.00^a^^,^^b^	0.61	10.22^a^^,^^b^	1.57	3.25^a^
having had sunburnt skin in childhood	81.5	1298	26.62^a^^,^^b^	30.96^a^^,^^b^	7.98^b^	12.97^a^^,^^b^	0.51	0.78
having a large number of moles on your skin	85.9	1396	15.50^a^^,^^b^	2.36	0.89	4.14	3.35^a^	3.05
having fair skin with freckles that burns easily	92.4	1594	5.44	4.90^b^	0.83	4.34	0.36	0.01
using sunbeds	82.4	1298	1.45	27.78^a^^,^^b^	2.85	5.90^b^	0.60	2.50^a^
Attitudes towards sun-exposure (% ‘agree’/‘strongly agree’)																
‘My skin will age more quickly if I spend time in the sun’	88.9	1603	22.13^a^^,^^b^	30.11^a^^,^^b^	5.33^b^	17.81^a^^,^^b^	0.48	6.12^a^^,^^b^
‘I'm concerned exposure to the sun/UV may give me skin cancer’	76.6	1395	6.37	5.96^a^^,^^b^	0.53	0.94	0.28	37.51^a^^,^^b^
‘A suntan makes me look attractive’	50.7	921	12.03^a^^,^^b^	6.95^b^	2.10	9.39^a^^,^^b^	1.27	35.57^a^^,^^b^
‘A suntan makes me look healthier’	66.3	1208	13.75^a^^,^^b^	0.85	6.86^b^	2.61	5.78^a^^,^^b^	21.92^a^^,^^b^
Behaviour (% who reported doing)																
using high factor sunscreen	36.6	676	49.80^a^^,^^b^	24.04^a^^,^^b^	3.21	19.34^a^^,^^b^	4.13^a^^,^^b^	45.14^a^^,^^b^
stay in the shade	36.6	677	27.18^a^^,^^b^	40.31^a^^,^^b^	0.81^a^	10.42^b^	1.20	79.76^a^^,^^b^
cover up	38.1	704	5.85	1.45	2.55	7.62^a^^,^^b^	2.93^a^	75.96^a^^,^^b^

a Significant in bivariate analysis.

b Significant in multiple regression analysis.

### Knowledge of protective behaviours

Respondents were most likely to know they should stay in the shade (65%), followed by covering up (50%). Seventy-seven percent of respondents mentioned using sunscreen, but less than half (43%) specified that the sunscreen needed to be high factor (SPF 15+).

Awareness of sun-safe behaviours was higher among those with educational qualifications. Women were more likely to know that people should use high factor sunscreen and stay in the shade to protect against skin cancer. Knowledge of staying in the shade, covering up and using high factor sunscreen was higher among older age groups. Having skin that burns easily was only associated with greater awareness of the need to cover up.

### Perceived importance of skin cancer risk factors

Awareness of known skin cancer risk factors was quite high. Sun-bathing without using suntan lotion was the most frequently endorsed risk factor (94%), followed by having fair skin that burns easily (92%) and getting sunburnt as an adult (88%). The perceived importance of burning during childhood was viewed as slightly less important (81.5%) and on a par with using sunbeds (82.4%). The factor rated least important in promoting skin cancer risk was sunbathing using suntan lotion (61.5%).

Women and those with educational qualifications were consistently more likely to endorse the risk factors. Those who reported burning easily in the sun were more likely to rate sunbathing, either with or without suntan lotion, as an important risk factor, but were no more likely to rate skin type as a risk factor.

Older people were more likely to believe that sunburn in childhood and having moles were risk factors for skin cancer.

### Attitudes about sun exposure

More participants endorsed the negative attitudes than the positive attitudes associated with sun exposure, with 89% agreeing with the statement that their skin would age more rapidly if they spent time in the sun and 77% agreeing with the statement that they were concerned sun exposure may give them skin cancer. Two-thirds agreed that a suntan made them look healthier and half agreed that a suntan made them look attractive.

Women were more likely to worry that sun exposure would cause their skin to age or lead to skin cancer than men, but there were no gender differences in positive attitudes. Both concern about the ageing effects of the skin and beliefs that a suntan made them look more attractive were higher among those with educational qualifications.

Older age groups were more likely to be concerned about the ageing effects of sun exposure and less likely to think a suntan made them look more attractive. However, they were more likely to think suntan made them look healthier.

Negative attitudes were higher among those who reported burning easily in the sun and positive attitudes were lower. The belief that a suntan made people look healthier was also higher among those who were married/living as married compared to those who were single, widowed or divorced.

### Practice of sun-protective behaviours

Reported use of sun-safe behaviours was low. Only one-third of participants said they used high factor sunscreen, sought shade or covered up in the sun. Women were more likely to report using high factor sunscreen and seeking shade than men, although there were no gender differences in covering up. Similarly, having educational qualifications was associated with a greater likelihood of using high factor sunscreen and seeking shade, but did not emerge as an independent predictor of covering up. Those with skin that burns easily were more likely to report doing all three of the sun-protective behaviours. The use of high factor sunscreen and seeking shade were both higher among older age groups. There was no significant association between age and covering up.

## Discussion

Knowledge of what to do to reduce skin cancer risk was modest. The behaviour identified most frequently was to avoid the sun by seeking shade; however, only two-thirds of people mentioned this. Fifty percent mentioned covering up. Although 77% of respondents mentioned using sunscreen (and this was the most frequent response to the question), less than half (43%) correctly specified that the sunscreen should be high factor. Consistent with previous research in this area, men and those without educational qualifications had poorer knowledge [e.g. (Campbell and Birdsell, 1994; Bourke and Graham-Brown, 1995; Paul *et al.*, 2003)], and such groups may need to be specifically targeted in future sun-awareness campaigns.

The majority of respondents recognized the importance of the skin cancer risk factors presented to them. However, there was evidence people overestimate the cancer-protective effect of sunscreen because a substantial proportion did not judge sunbathing *with* sunscreen to be a significant risk factor. There was also evidence that people may underestimate the importance of sunburn in the aetiology of skin cancer. Only 40% rated getting sunburnt as either an adult or as a child as a ‘very’ important risk factor. Again, both men and those with lower levels of educational qualifications were consistently less likely to rate all items as important skin cancer risk factors. Worryingly, those who reported burning easily in the sun were no more likely to know that skin type was a risk factor for skin cancer, suggesting they are no more aware of their increased personal risk than those with lower risk skin types.

A number of studies have examined knowledge of skin cancer risk factors, but sampling differences limit the comparisons that can be made with the results of the present study [e.g. (Newman *et al.*, 1996; Balanda *et al.*, 1999; Livingston *et al.*, 2001)]. One population representative telephone survey conducted by the American Academy of Dermatology (ADD) in 1995 found lower levels of awareness of risk factors compared with the present study (US Department of Health and Human Services, 1996). For example, in the US survey only 58% mentioned severe childhood sunburn was a risk factor for melanoma compared with the 82% who endorsed it here, but this may simply reflect the methodological differences between the studies: the ADD study looked at awareness of the risk factors for melanoma rather than skin cancer in general and it is unclear whether they used prompted or unprompted recall. If unprompted recall was used, then the lower levels of recall are unsurprising because this method consistently leads to lower levels of apparent awareness compared with prompted recall (the method used in the present study) (Waller *et al.*, 2004).

There were higher ratings overall for the negative attitude statements than the positive attitude statements about sun exposure. Despite this, only one-third reported actually using sunscreen, covering up or seeking shade to reduce their skin cancer risk. Comparisons with previous studies are again limited because of methodological differences. For example, some studies have used direct observation rather than self-reported behaviour (Hill and Boulter, 1996) and have asked about behaviour on particular days at set times (Hill *et al.*, 1993; Campbell and Birdsell, 1994) rather than usual behaviour.

Consistent with previous research, the present study showed that women reported they were more likely to use sunscreen and seek shade in an effort to protect themselves from the sun than men (Hill *et al.*, 1993; Arthey and Clarke, 1995; Schofield *et al.*, 2001). However, no gender difference was found in covering up. Previous evidence of gender differences in the use of protective clothing has been mixed. A population representative sample of white Americans found no gender differences in use of protective clothing (Hall *et al.*, 1997); however, some studies have reported that men are more likely to use sunhats than women [e.g. (Hill *et al.*, 1993; Campbell and Birdsell, 1994)] and the higher rates of skin cancer on the torso in men suggests they are less likely to cover their upper body in the sun than women [Marks *et al.*, 1993; cited in (Arthey and Clark, 1995)].

Age and educational level have also been associated with particular methods of sun protection (Arthey and Clarke, 1995). In the present study there were no age differences in reported use of covering up, but older people were more likely to report seeking shade and younger groups more likely to say they use high factor sunscreen. Possession of educational qualifications was associated with greater use of high factor sunscreen and seeking shade, and less use of covering up (although this latter result was not an independent effect). However, it should be noted that interactions between sociodemographic variables and use of different sun-protective behaviours were not formally assessed.

The results of the present study demonstrate that both increases in knowledge about appropriate sun-safe behaviour and changes in attitudes towards suntans are needed if behaviour is to change (Hill *et al.*, 1984; Wesson and Silverberg, 2003). Future sun-awareness campaigns need to highlight the role of burning in skin cancer, in both childhood and adulthood, as these risk factors were among the least frequently endorsed. Also, a substantial proportion of the population did not consider sunbathing using sunscreen to be a risk factor for skin cancer. Awareness of the limitations of sunscreen and the need to use other sun-protective measures, such as covering up, are required if skin cancer incidence is to be reduced.
